# Resveratrol Encapsulation and Release from Pristine and Functionalized Mesoporous Silica Carriers

**DOI:** 10.3390/pharmaceutics14010203

**Published:** 2022-01-16

**Authors:** Simona Ioniţă, Daniel Lincu, Raul-Augustin Mitran, Laila Ziko, Nada K. Sedky, Mihaela Deaconu, Ana-Maria Brezoiu, Cristian Matei, Daniela Berger

**Affiliations:** 1 Faculty of Applied Chemistry and Material Science, University “Politehnica” of Bucharest, 1–7 Polizu Street, 011061 Bucharest, Romania; ionitasimona05@gmail.com (S.I.); daniel.lincu1113a@gmail.com (D.L.); mihaela_deaconu@yahoo.com (M.D.); anamariabrezoiu@gmail.com (A.-M.B.); cristian.matei@upb.ro (C.M.); 2 “Ilie Murgulescu” Institute of Physical Chemistry, Romanian Academy, 202 Splaiul Indepedenței, 060021 Bucharest, Romania; 3 School of Life and Medical Sciences, University of Hertfordshire, hosted by Global Academic Foundation, New Administrative Capital, Cairo 11865, Egypt; laila.adel@aucegypt.edu (L.Z.); nadasedky22@gmail.com (N.K.S.); 4 Biology Department, School of Sciences and Engineering, American University in Cairo, Cairo 11835, Egypt

**Keywords:** mesoporous silica, resveratrol, drug delivery, kinetics, functionalization, cell cycle analysis

## Abstract

Resveratrol, a naturally occurring polyphenol, has attracted significant attention due to its antioxidant, cardioprotective and anticancer potential. However, its low aqueous solubility limits resveratrol bioavailability and use. In this work, different mesoporous silica matrices were used to encapsulate the polyphenol and to increase its dissolution rate. Pristine MCM-41, MCM-48, SBA-15, SBA-16, FDU-12 and MCF silica were obtained. The influence of SBA-15 functionalized with aminopropyl, isocyanate, phenyl, mercaptopropyl, and propionic acid moieties on resveratrol loading and release profiles was also assessed. The cytotoxic effects were evaluated for mesoporous carriers and resveratrol-loaded samples against human lung cancer (A549), breast cancer (MDA-MB-231) and human skin fibroblast (HSF) cell lines. The effect on apoptosis and cell cycle were assayed for selected resveratrol-loaded carriers. The polyphenol molecules are encapsulated only inside the mesopores, mostly in amorphous state. All materials containing either pristine or functionalized silica carriers increased polyphenol dissolution rate. The influence of the physico-chemical properties of the mesoporous carriers and resveratrol–loaded supports on the kinetic parameters was identified. Resv@SBA-15-SH and Resv@SBA-15-NCO samples exhibited the highest anticancer effect against A549 cells (IC_50_ values were 26.06 and 36.5 µg/mL, respectively) and against MDA-MB-231 (IC_50_ values were 35.56 and 19.30 µg/mL, respectively), which highlights their potential use against cancer.

## 1. Introduction

One of the most promising health care application of nanomaterials lies in the development of controlled drug delivery systems. These systems can both increase the effectiveness and decrease side effects of biologically active compounds [1]. Mesoporous silica nanoparticles (MSN) are promising matrices for drug delivery systems [2,3,4]. MSN contain mesopores of 2–50 nm, resulting in materials with high porosity, with specific surface areas up to 1000 m^2^/g and pore volumes of 0.5–3 cm^3^/g, which is important for the adsorption of guest molecules. The mesopores typically have low polydispersity and an ordered arrangement [5,6]. The mesoporous silica matrices are biocompatible, and they can be obtained in bulk through chemical synthesis [7]. One of the most important features of mesoporous silica is the easy way to modify their properties through chemical synthesis. In addition to tailoring the textural features, mesopore size and arrangement through specific synthesis conditions and reagents, the silica framework can be doped with ad-atoms, while the surface silanol groups can be functionalized with various organic moieties [8,9,10,11,12].

Many biologically active compounds exhibit reduced aqueous solubility, which limits their bioavailability. Mesoporous silica encapsulation was shown to increase both the solubility and the dissolution rate of low-solubility compounds [13], such as carbamazepine [14], ibuprofen [15] or aminoglutethimide [16]. 

Resveratrol (3,5,4′-trihydroxy-*trans*-stilbene, Resv) is a small organic compound with low aqueous solubility which occurs naturally in plants. This compound is secreted by plants in response to certain stressors and it possesses antioxidant, anti-inflammatory, antiaging, cardioprotective and anti-cancer effects [17]. Resveratrol exists as two isomers: *cis* and *trans*, the last one being biologically more active. Despite the low aqueous solubility (0.3 mg/L), it exhibits high membrane permeability. However, the therapeutic application of resveratrol remains limited because of its short biological half-life and rapid metabolism and elimination [18]. The bioavailability of biologically active compounds is widely thought to be determined by their aqueous solubility, membrane permeability and metabolic stability. Lately, nano-formulations of resveratrol based on polymeric nanoparticles, cyclodextrins, micelles, liposomes, mesoporous silica etc. have been focused on enhancing its aqueous solubility and stability, and thus, the bioavailability [19,20,21]. For instance, liposomes prevent the isomerization of *trans*-resveratrol, being found that 70 wt.% of encapsulated polyphenol molecules remained as *trans*-isomer after UV light exposure in comparison with only 10 wt.% when free resveratrol was irradiated in the same conditions [22]. Zu et al. used carboxymethyl chitosan (CMCS) for encapsulation of resveratrol in amorphous state. This nano-formulation exhibited an improved aqueous solubility and thus, an enhanced in vivo bioavailability than free resveratrol [23]. Juère et al. showed that the aqueous solubility of encapsulated resveratrol in MCM-48 silica matrices with a 20 wt.% polyphenol content is higher than that of the crystalline free biologically active compound regardless the diameter of silica nanoparticles and this phenomenon was explained through crystalline/amorphous character of the free/encapsulated resveratrol [21].

Mesoporous silica can be used to encapsulate resveratrol to increase its solubility and dissolution rate. Mobil Composition of Matter No. 41 (MCM-41), Korea Advanced Institute of Science and Technology-6 (KIT-6) [24], MCM-48 [21,25], MCM-41, Santa Barbara Amorphous-16 (SBA-16) [26], and SBA-15 [27] mesoporous silica materials were used as resveratrol carriers. For instance, it was observed that resveratrol encapsulated in MCM-48 spherical nanoparticles exhibited more than 5-fold higher permeability coefficient than a resveratrol suspension on human colon carcinoma CaCo-2. The permeability coefficient of a drug or nanoparticles correlates with the level of absorption in vivo. Also, at low tested concentration—5 μg/mL—resveratrol-loaded MCM-48 silica demonstrated a higher anti-inflammatory activity compared to both resveratrol suspension and solution [21]. Mesoporous silica nanoparticles have also been employed and the effect of functionalization with amino or phosphonate groups has been studied [28]. Complex drug delivery systems containing mesoporous silica nanoparticles with small mesopores (<5 nm) have also been obtained and used to load resveratrol, particularly for anti-cancer applications [29]. However, mesoporous silica with larger pore sizes between 10 and 30 nm, such as mesocellular foams, have not been studied so far. 

The aim of the current work is to provide a comprehensive study of the influence of the textural parameters and functional groups grafted on silica surface on the resveratrol release kinetics. Mesoporous silica with larger pore size, such as Fudan University-12 (FDU-12) and mesocellular foam silica (MCF) was used as carriers for the target compound for the first time. The adsorption and release of resveratrol from these carriers is compared to that from MCM-41, SBA-15, MCM-48, and SBA-16 samples possessing hexagonal or cubic mesopore arrangement. Furthermore, the comparative effects of functionalization with organic groups containing different moieties have not been reported so far. Thus, mesoporous silica matrices functionalized with 3-aminopropyl, isocyanate, phenyl, 3-mercaptopropyl, and propionic acid were obtained starting from pristine SBA-15 silica. The novel formulations, as well as the reference formulations were tested for cytotoxicity against A549 non-small cell lung cancer and MDA-MB-231 triple negative breast cancer cell lines. In addition, the two most potent anticancer formulations against A549 cells line were selected for further investigation of the biological activity through apoptosis assays and cell cycle analysis.

## 2. Materials and Methods

### 2.1. Materials and Reagents

MCM-41 hexagonal structured silica, 36.5–38 wt.% hydrochloric acid, poly(ethylene glycol)-block-poly(propylene glycol)-block-poly(ethylene glycol) (Pluronic P123, average molecular weight 5800), poly(ethylene glycol)-block-poly(propylene glycol)-block-poly(ethylene glycol) (Pluronic F127, average molecular weight 12,600), potassium hydroxide, 98% sulfuric acid, sodium chloride, 1,3,5-trimethylbenzene (TMB), toluene, ethanol were purchased from Sigma-Aldrich (Merck Group, Darmstadt, Germany), 25 wt.% ammonia aqueous solution from Scharlau (Scharlab S.L., Barcelona, Spain), trimethylhexadecylammonium bromide (CTAB, Alfa Aesar, Ward Hill, MA, USA), and tetraethyl orthosilicate, (TEOS, Fluka, Seelzer, Germany). All organosilanes: (3-aminopropyl)triethoxysilane (APTES, 95%), (2-cyanoethyl)triethoxysilane (CETES, 97%), (3-isocyanatopropyl)triethoxysilane (ICPTES, 95%), triethoxyphenylsilane (TEPS, 98%), (3-mercaptopropyl)triethoxysilane (MPTES, 95%), and toluene (anhydrous, 99.8%) were acquired from Sigma-Aldrich and used without further purification. Resveratrol (≥99% purity) was purchased from Evolva SA (Reinach, Switzerland), and ultrapure deionized water (Millipore Direct-Q3UV with Biopack UF cartridge, Merck Group, Darmstadt, Germany) was used in all experiments.

### 2.2. Synthesis of Pristine Mesoporous Carriers

The syntheses of the mesoporous silica carriers were carried out according to established procedures, by sol-gel method in the presence of various surfactants and additives.

The synthesis of SBA-15 mesoporous silica was performed by a reported sol-gel method [30]. 2.5 g of Pluronic P123 were dissolved under vigorous stirring at room temperature in a beaker containing 13.8 mL concentrated hydrochloric acid and 79 mL distilled water. Afterwards, 5.9 mL of TEOS were added and the mixture was stirred at 40 °C for 24 h. The resulting sol was aged statically at 100 °C for 24 h under autogenerated pressure in a Teflon-lined autoclave. The solid phase was recovered by filtration and intensively washed with distilled water and ethanol. Lastly, the dried white particles were calcined at 550 °C for 5 h in static air. A molar ratio TEOS:Pluronic P123:HCl:H_2_O of 1:0.016:6.3:185 was employed. 

The synthesis of MCM-48 silica was performed by dissolving 9.675 g CTAB in an alkaline solution containing 1.143 g of KOH in 45.6 mL water. To the surfactant solution, 9.185 mL of TEOS were added and the reaction mixture was stirred at 35 °C for 24 h. The resulting sol was transferred to a Teflon-lined autoclave and aged at 120 °C for 24 h under autogenerated pressure. The resulting solids were filtered off and washed with water and ethanol. The as-prepared MCM-48 was calcined at 550 °C for 5 h in static air. This material was denoted “MCM-48C”.

The synthesis of SBA-16 cubic silica was performed according to our previously reported protocol [31]. FDU-12 cubic silica was obtained by a modification of the literature protocol [32]. Thus, 2.0 g Pluronic F127, 2.75 mL TMB and 3.93 g NaCl were dissolved into a solution containing 20 mL 37% HCl and 100 mL water to whom 8.83 mL TEOS were added and then the reaction mixture was stirred at 40 °C for 24 h, being afterwards transferred to a Teflon-lined autoclave for static hydrothermal treatment at 100 °C for 72 h. The resulting solid was filtered off, washed, and calcined at 550 °C for 5 h.

The mesocellular foam silica (MCF) was obtained using the same protocol as for the SBA-15 synthesis, except for adding 3.18 mL TMB in the initial Pluronic P123 solution. The solid was obtained at a molar ratio TEOS:P123:TMB:HCl:H_2_O of 1:0.016:0.864:6.3:185.

### 2.3. Synthesis of Functionalized SBA-15 Materials

The functionalization of the mesoporous silica was carried out in an anhydrous toluene as solvent and different organosilanes as organic functional groups source. Pristine SBA-15 silica was dried under vacuum at 110 °C prior to use. A typical functionalization reaction was performed as follows: 1 g silica was added into 100 mL toluene together with the organosilane, in an organosilane/SBA-15 molar ratio of 1/5. The reaction mixture was heated under reflux for 20 h. The functionalized materials were recovered by centrifugation and washed with toluene, acetone, and ethanol. In the case of aminopropyl functionalized sample, an additional washing with HCl 0.1 M was necessary for removing some of the polycondensed amine groups [33]. The resulting solids were dried at room temperature for 24 h. The propionic acid functionalized silica was obtained from corresponding cyanoethyl functionalized SBA-15 material through hydrolysis in strong acidic medium. 1 g of cyanoethyl functionalized silica, prepared using CETES as described above, was dispersed in 30 mL 50% sulfuric acid, and heated at 120 °C for 16 h. The resulting material was washed with water until neutral pH. The SBA-15 materials functionalized with aminopropyl, isocyanate, phenyl, propanethiol, cyanoethyl and propylcarboxylic acid are denoted SBA-15-NH_2_, SBA-15-NCO, SBA-15-Ph, SBA-15-SH, SBA-15-CN, and SBA-15-COOH. 

### 2.4. Resveratrol Loading and In Vitro Release

The bioactive substance was loaded by incipient wetness impregnation method. A 25 mg/mL resveratrol ethanolic solution was added to the silica carriers followed by homogenization and drying under vacuum. A theoretical 20% (wt.) resveratrol was considered for all samples. The resveratrol-loaded samples are denoted Resv@*carrier*, where carrier represents the mesoporous silica matrix. The resveratrol release experiments were performed in simulated intestinal fluid, using phosphate buffer solution (PBS) pH = 6.8, at 37 °C and constant magnetic stirring at 150 RPM. The materials were added to a dialysis membrane together with 1 mL PBS and the concentration of resveratrol in the release medium was measured using UV-Vis spectrometry. The resveratrol release profiles were obtained using 5 mg of polyphenol-loaded sample in 50 mL of PBS medium.

### 2.5. Cell Culture Conditions

Cells were grown in the same culture conditions of DMEM media with 10% FBS (Gibco, Thermo Fisher Scientific-Waltham, MA, USA) under the standard cell culture conditions of 5% CO_2_ and temperature of 37 °C. In this study, the adenocarcinoma A549, as well as the triple negative breast cancer MDA-MB-231 cell lines were used, in addition to the normal human skin fibroblast HSF cell line.

#### 2.5.1. Cytotoxicity Assay

The Sulforhodamine B (SRB) assay was employed to assess the cytotoxicity of all the tested formulations in addition to the original polyphenol on human lung cancer (A549), breast cancer (MDA-MB-231) and human normal skin fibroblast (HSF) cell lines. A volume of 100 μL cell suspension containing 5 × 10^3^ cells in complete culture media was added to each well of a 96-well plate for 24 h. The tested bioactive compound or formulations were dispersed in PBS and diluted with DMEM media. Afterwards, a volume of 100 μL media containing formulations or drugs was added to the cells at different concentrations. 0.05, 0.5, 5, 50 and 500 μM concentrations were used for the resveratrol-loaded samples, while 0.01, 0.1, 1, 10 and 100 μM concentrations were employed for resveratrol. After 72 h of treatment, culture media was removed thoroughly and replaced with 150 μL of 10% trichloroacetic acid (TCA). The culture plate was left at 4 °C for 1 h to fix the cells. This was followed by removal of the TCA solution and five consecutive wash steps with distilled water. A volume of 70 μL SRB solution (0.4% w/v) was placed in each well with successive incubation in a dark room for 10 min. Subsequently, 3 wash steps were performed using 1% acetic acid and plates were left to air-dry overnight. Finally, an aliquot of 150 μL of Tris (10 mM) was added to each well and the absorbance was recorded at 540 nm using a BMG LABTECH^®^-FLUOstar Omega microplate reader (Ortenberg, Germany).

#### 2.5.2. Apoptosis Assay

The apoptosis assay was performed using Annexin V-FITC apoptosis detection kit (Abcam Inc., Cambridge, UK) conjoined with two fluorescent channel flow cytometry. The assay was done as reported earlier [34,35]. Two formulations were selected for this assay, namely Resv@SBA-15-SH and Resv@SBA-15-NCO, as they had the lowest IC50 values against the adenocarcinoma A549 cells. The test formulations were applied to the A549 cells and left in the incubator for 48 h. An estimate of 10^5^ cells were obtained by trypsinization and washed twice with ice-cold PBS (pH 7.4). To stain the cells, a volume of 0.5 mL of Annexin V-FITC/PI solution was applied and then the cells were kept for 30 min away from light at standard room temperature. Directly afterwards, cells were placed in the Novocyte™ ACEA flow cytometer (ACEA Biosciences Inc., San Diego, CA, USA) where FL2 and FL1 detectors were selected for determination of PI and FITC signals, respectively. An acquisition of 12,000 events was implemented per sample. The ACEA NovoExpress™ software (ACEA Biosciences Inc., San Diego, CA, USA) was employed in the calculations and quadrant analysis.

#### 2.5.3. Cell Cycle Analysis

The cell cycle analysis was performed as previously described [34]. Resv@SBA-15-SH and Resv@SBA-15-NCO were selected for this assay. The samples were first added to the A549 cells and left in the CO_2_ incubator for 48 h. The cells were first trypsinized to obtain sufficient cell count (~10^5^ cells), then washed with phosphate buffer saline (PBS). Then PBS washes ensued, with a final resuspension step was conducted using ethanol (60%) at 4 °C. Subsequently, a volume of 1 mL PBS encompassing 50 μg mL^−1^ RNAase A and 10 μg mL^−1^ propidium iodide was added to re-suspend the cells. The cell suspension was then incubated at 37 °C for in the dark for 20 min duration. Lastly, the Novocyte™ ACEA flow cytometer was used to conduct the cell cycle analysis of the provided cell suspension. The DNA content of the cells were then assessed with the help of FL2 signal detector of the flow cytometer. An estimate of 12,000 events was acquired per sample. The frequency of cells in each of the cell cycle phases was analyzed using the ACEA NovoExpress^TM^ software.

### 2.6. Characterization of Materials

Small-angle and wide-angle X-ray diffraction (XRD) were performed using a Rigaku Miniflex II diffractometer (Rigaku Corporation, Tokyo, Japan) with CuKα (λ = 1.5406 Å) radiation. Fourier transform infrared (FTIR) spectroscopy measurements were carried out on a Bruker Tensor 27 spectrometer (Bruker Corporation Optik GmbH, Bremen, Germany) using KBr pellets. Nitrogen adsorption-desorption isotherms were recorded on a Quantachrome Autosorb iQ2 surface and porosity analyzer (Quantachrome Instruments, Boynton Beach, FL, USA) at 77 K. Specific surface areas were computed using the Brunauer–Emmett–Teller (BET) method in the 0.1–0.3 relative pressure range of the adsorption branch of the isotherm. The total pore volume was calculated at 0.98 relative pressure. The pore size distribution curves were evaluated using the Barrett Joyner Halenda (BJH) method on either the adsorption or desorption branches of the isotherm. Thermogravimetric analyses (TG) were acquired in the 25–1000 °C temperature range using a Mettler Toledo TGA/SDTA851e (Mettler Toledo, Greifensee, Switzerland) instrument under 80 mL min^−1^ synthetic airflow using open alumina crucibles and a heating rate of 10 °C/min. Differential scanning calorimetry (DSC) measurements were carried out using a Mettler Toledo DSC 3 calorimeter under 80 mL/min nitrogen flow, at a heating rate of 10 °C/min. The samples were placed inside sealed aluminum crucibles with pierced lids and measured between 25 and 280 °C, without any prior thermal treatment. UV-VIS spectra were recorded on an Agilent Cary 60 spectrometer (Agilent Scientific Instruments, Santa Clara, CA, USA) and scanning electron microscopy (SEM) was performed on a Tescan VEGA 3 LM (Tescan, Brno, Czech Republic). Transmission electron microscopy (TEM) was performed on a FEI (Hillsboro, OR, USA) Tecnai G2-F30 S-Twin field-emission gun scanning transmission electron microscope (FEG STEM) operating at 300 kV. A drop of the mesoporous silica alcoholic suspension was mounted on a holey carbon film copper grid allowing the solvent to evaporate at room temperature. The statistical analysis of the drug release process was performed using the Analysis Toolpak in Microsoft Excel. 

## 3. Results

### 3.1. Physico-Chemical Characterization of the Mesoporous Carriers and Resveratrol–Loaded Samples

A series of pristine mesoporous silica materials, denoted as MCM-48C, MCM-41, FDU-12, MCF, SBA-16, and SBA-15, with varying pore size and arrangement was obtained. Furthermore, various organic groups such as 3-aminopropyl, isocyanate, phenyl, 3-mercaptopropyl, and propionic acid moieties were grafted onto SBA-15 silica resulting following carriers: SBA-15-NH_2_, SBA-15-NCO, SBA-15-Ph, SBA-15-SH, and SBA-15-COOH. The effect of these properties on the release of the biological active molecule, *trans*-resveratrol (Resv.) from polyphenol-loaded samples, labelled as Resv@*carrier*”, was investigated. 

**Figure 1:**
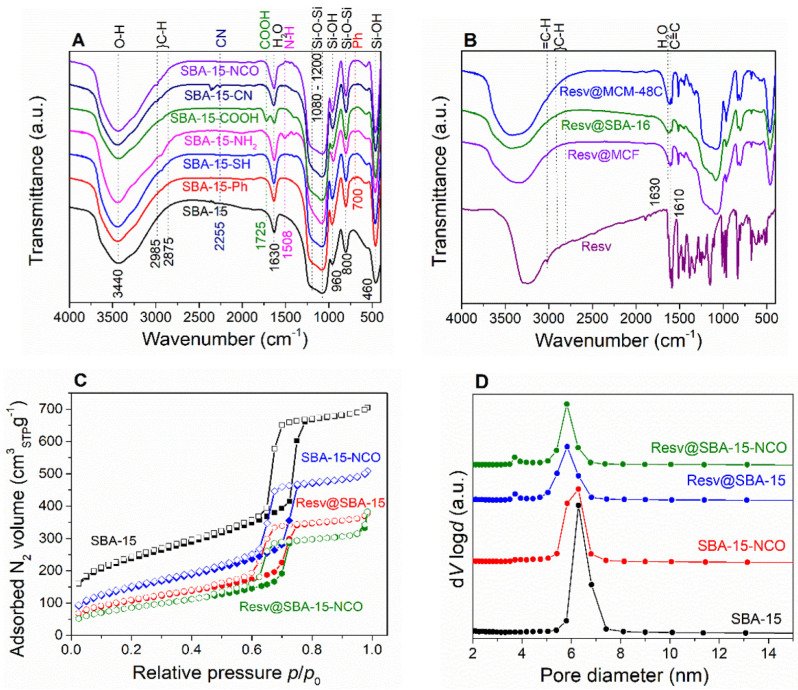
FT-IR spectra of pristine and functionalized SBA-15 (**A**) and resveratrol–loaded pristine silica carriers (**B**); representative nitrogen adsorption-desorption isotherms of pristine and functionalized SBA-15 carriers and corresponding resveratrol-loaded carriers (**C**) and pore size distribution curves of the same selected SBA-15-type carriers and resveratrol-loaded materials (**D**).

Infrared spectroscopy (FTIR) was applied to reveal the presence of different chemical species and functional groups. The characteristic vibrations of the pristine silica, symmetric and asymmetric Si–O–Si stretching (800 cm^−1^ and 1080 cm^−1^, respectively), symmetric Si–O bending (460 cm^−1^) and Si–OH stretching vibrations (960 cm^−1^) can be noticed for all samples (Figure 1A,B). The broad O–H stretching vibrations, centered at 3430 cm^−1^ and the physiosorbed water characteristic vibrations at 1630 cm^−1^ are also present in all spectra. FTIR spectroscopy also confirms the presence of the specific vibrations associated with the functional organic groups (Figure 1A). The presence of specific organic groups for the functionalized carriers is also be evidenced by FTIR spectroscopy (Figure 1A). The C–H stretching vibrations (2875–2985 cm^−1^) can be noticed for all functionalized samples containing aliphatic –CH_2_– moieties. The characteristic C= O stretching vibrations (1725 cm^−1^) and the N–H bending vibrations (1508 cm^−1^) were noticed for carboxyl and amino-functionalized samples, respectively. The SBA-15-Ph carrier exhibits the characteristic C–H bending vibration of the aromatic ring, (700 cm^−1^) while the CN stretching vibration (2260 cm^−1^) can be noticed for SBA-15-CN, which disappears in the case of SBA-15-COOH that was obtained from SBA-15-CN material (Figure 1A).

The porosity of all samples was investigated by nitrogen adsorption–desorption isotherms (Figure 1C; Figure S1, Supplementary Information). All mesoporous carriers and resveratrol-loaded samples exhibit type IV isotherms with hysteresis between the adsorption and desorption curves, which indicates the existence of mesopores. MCF and Resv@MCF have the highest adsorbed N_2_ volume and total pore volume, as well as H2(b) hysteresis. FDU-12 and SBA-16 present H2(a) hysteresis loops characteristic for materials with cubic mesophase arrangement and “ink-bottle” pores [36]. MCM-48C and MCM-41 exhibit H4 hysteresis, indicating mesopores with diameters lower than 3.8 nm. All SBA-15-type samples show H1 hysteresis, characteristic for materials with narrow, unblocked mesopores. The specific surface area, total pore volumes and average pore diameters were computed from the isotherm (Table 1). The pore size distribution curves were determined with Barrett Joyner Halenda model (Figure 1D, Figure S2, Supplementary Information). The average pore diameters calculated from the desorption and adsorption branches of the isotherm correspond to the size of the larger spherical cells and smaller window pores, respectively, for the carriers with “ink-bottle”-type mesopores (FDU-12, SBA-16, and MCF). 

The content of functional groups grafted on the silica pore walls surface and the amount of resveratrol-loaded on silica-type carriers were determined through thermogravimetric analyses (TGA) performed in air considering the mass loss difference between the pristine and functionalized carriers and the resveratrol-loaded supports (Table 1). 

**Figure 2:**
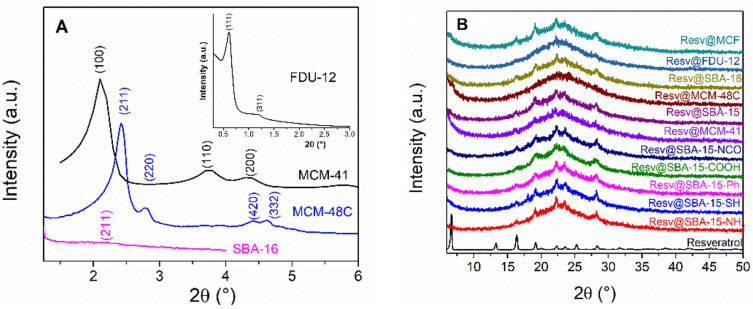
Small–angle XRD patterns of mesoporous silica carriers (**A**) and wide-angle XRD patterns of resveratrol loaded carriers (**B**).

Small-angle X-ray diffraction (XRD) was used to characterize the mesopore array of the silica matrices. The XRD patterns of MCM-41, SBA-15 and functionalized SBA-15 carriers exhibit the characteristic peaks associated with the (100), (110) and (200) Bragg reflections of the *P6mm* hexagonal space group (Figure 2A, Figure S3). The diffraction peaks of the MCM-48C sample could be indexed as the (211), (220), (420) and (332) reflections belonging to the body centered cubic *Ia3d* space group. The (211) peak of the same space group can hardly be noticed for SBA-16, indicating a less-ordered mesopore array for this carrier. Finally, two small-angle diffraction peaks, indexed as the (111) and (311) reflections of the face centered cubic *Fm3m* space group can be noticed in the case of the FDU-12 carrier (Figure 2A-inset). 

The wide-angle XRD patterns of resveratrol-loaded samples show the presence of a broad peak between 15–30° 2*θ*, which is characteristic for amorphous silica (Figure 2B). all patterns are superimposed with several peaks corresponding to crystalline resveratrol. 

**Figure 3:**
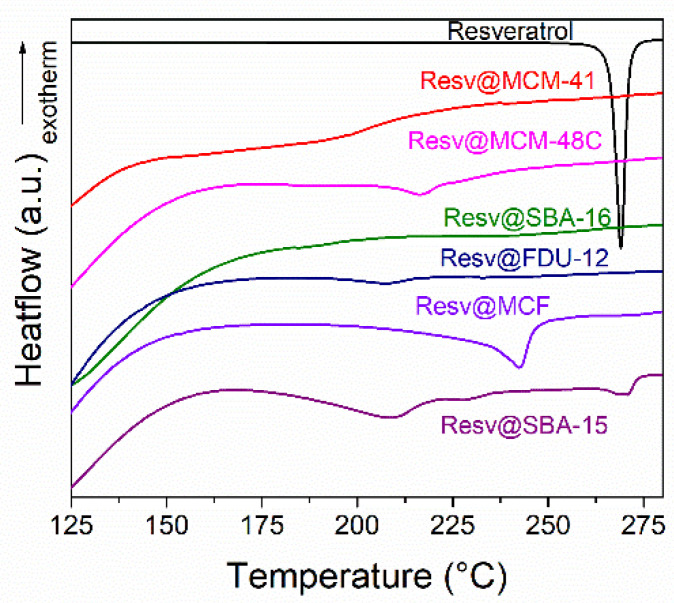
DSC traces of resveratrol and resveratrol-loaded pristine silica carriers.

Differential scanning calorimetry (DSC) analyses were performed to assess the distribution of crystalline resveratrol between the mesopores and interparticle space (Figure 3, Figure S4, Supplementary Information). This method is based on the melting point decrease due to size reduction in the nanometer range [37]. The melting point depression follows an inverse law relationship with respect to particle size, quantified by the Gibbs–Thompson equation (Equation (1)) [38]:

(1) $$\Delta T=-\frac{T\left(\infty \right)M\gamma_{sl}}{\rho \Delta H_{f}}\frac{\alpha }{r}$$

where ΔT=T(∞)−T(r) is the melting point depression due to nanoconfinement, i.e., the difference between the melting points of bulk, T(∞), and nanoconfined phase of radius r, T(r), $\Delta H_{f}$ represents the heat of fusion,$\;\gamma_{sl}$ is the surface tension of the solid-liquid interface, ρ represents the density, M denotes the molar mass and α is a parameter characterizing pore geometry.

Pure resveratrol melts at 266 °C with a heat of fusion of ~250 Jg^−1^. The resveratrol-loaded silica-type carriers however exhibit significant lower melting points, in the 178–204 °C temperature range. All resveratrol-loaded pristine mesoporous silica carriers exhibit low heat of fusion values, between −1 and −14 Jg^−1^ (Table 2).

The average crystallite size of resveratrol for resveratrol-loaded samples with a higher content of crystalline phase than 20% was computed from the wide–angle XRD data using Rigaku PDXL software version 4.0, which is based on the Sherrer equation: $Crystallite\;size=\frac{0.9*\lambda }{\beta *\cos\left(\theta \right)}$, where *β* represents the full width at half maximum. The crystallite size of resveratrol for all samples is lower than the pore size of the used carriers (Table 1 and Table 2), which is consistent with the presence of a crystalline phase of biologically active compound inside the silica mesopores.

**Figure 4:**
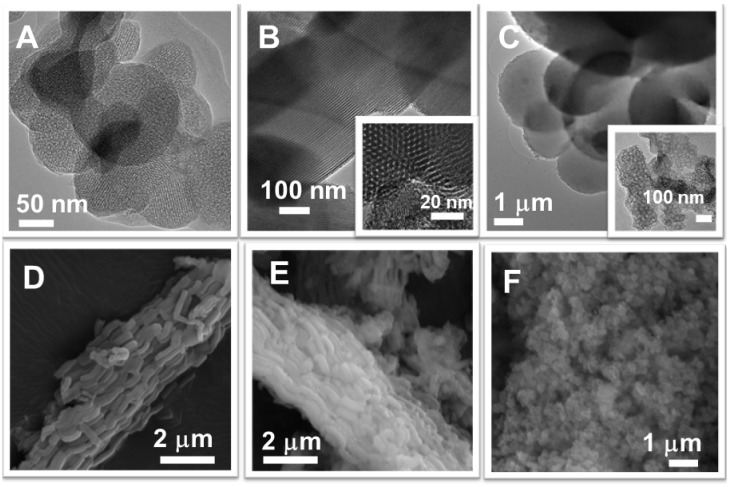
TEM images of MCM-48C (**A**), SBA-15 (**B**) and MCF (**C**) and SEM micrographs of SBA-15 (**D**), SBA-15-SH (**E**) and SBA-16 (**F**) carriers.

The morphology of the mesoporous silica carriers was assessed by transmission and scanning electron microscopy (Figure 4). The TEM investigation of MCM-48C and SBA-15 samples confirmed their ordered pore array with cubic symmetry (Figure 4A) and with long channels formation, respectively (Figure 4B) in agreement with small-angle XRD data. MCM-48C and MCF materials present spherical particles, ranging from 150 to 450 nm for MCM-48C (Figure 4A) and particles with bigger diameter, in the range of 1.5–3.5 µm (Figure 4C) and larger pore size in the case of MCF sample (Figure 4C-inset). SBA-16 sample has spherical nanosized-particles (in the range of 200–400 nm) with a narrow size distribution (Figure 4F). All SBA-15-type supports present rod-like particles with a length between 1.5 and 2.5 µm and an average dimensions ratio of 6. The functionalization of SBA-15 silica did not alter the morphology of particles (Figure 4C,E).

### 3.2. In Vitro Release Profiles and Kinetics Model

The delivery profiles of resveratrol-loaded carriers and the dissolution of pure resveratrol were obtained in simulated intestinal fluid, phosphate buffer solution pH 6.8, at 37 °C. A concentration of 20 μg/mL of bioactive compound was considered for the dissolution or release profiles. Because *trans*-resveratrol undergoes degradation under light, all experiments were performed in dark conditions.

**Figure 5:**
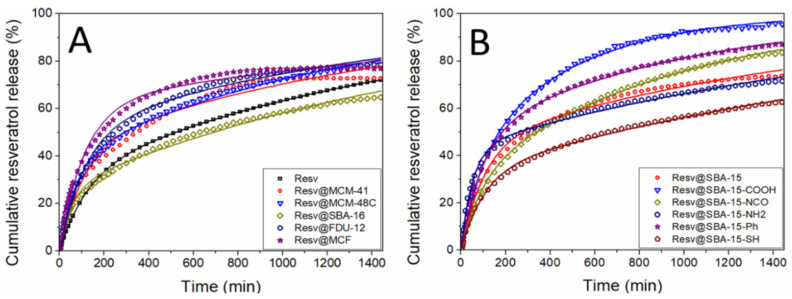
Resveratrol release profiles fitted with a three-parameter model for samples containing (**A**) pristine and (**B**) functionalized silica matrices.

Firstly, the cumulative release of resveratrol from the pristine silica carriers was compared with the dissolution of the same amount of solid biological active substance up to 24 h (Figure 5). All release profiles are characterized by a faster initial release up to 200–400 min, followed by a region of slower increase in resveratrol concentration. The fast initial release is usually denoted as the “burst” release region, while the following slower release rates constitute the “sustained” release regime. The burst release rates from all silica matrices are higher than the dissolution of free resveratrol. All composite samples exhibit increased resveratrol dissolution rate with respect to the free substance. 

The resveratrol release profiles from the functionalized SBA-15 supports present also the burst and sustained release regions (Figure 5B). Higher cumulative release is obtained after 24 h for samples except for Resv@SBA-15-SH in comparison with free resveratrol. Most functional groups except aminopropyl and 3-mercaptopropyl have resulted in higher total resveratrol release than the sample containing pristine SBA-15. The highest drug cumulative release is achieved by the Resv@SBA-15-COOH sample, which exhibits 96.8 ± 0.46 wt.% biological active substance delivered after 24 h.

The experimental release profiles were fitted with a theoretical three-parameters kinetic model proposed by Zeng et al. [39]. The model assumes that all biological active molecules are either strongly or weakly bound to the silica pore surface at the beginning of the release experiment. There is an equilibrium between the strongly bound (adsorbed) molecules and the weakly bound (desorbed) molecules, characterized by the molecular free energy, Δ*G*, parameter. The resveratrol adsorption and desorption are assumed to follow 1st order kinetics, with the *k_ads_* and *k_des_* constants, respectively. The desorbed molecules can be transported into the release medium by diffusion and convection processes, which are also assumed to follow 1st order kinetics, characterized by the *k_tr_* kinetic constant. The rate equation (Equation (2)) describing the cumulative release amount at time *t* can be written as follows:

(2) $$\frac{m\left(t\right)}{m\left(0\right)}=\frac{\lambda_{2}\left(k_{tr}-\lambda_{2}\right)}{\left(k_{ads}+k_{des}\right)\left(\lambda_{1}-\lambda_{2}\right)}\left(1-e^{-\lambda_{1}t}\right)+\frac{\lambda_{1}\left(\lambda_{1}-k_{tr}\right)}{\left(k_{ads}+k_{des}\right)\left(\lambda_{1}-\lambda_{2}\right)}\left(1-e^{-\lambda_{2}t}\right)$$

where m(t)/m(0) is the cumulative fraction of resveratrol released up to time t and $\lambda_{1,2}=\left[\left(k_{tr}+k_{ads}+k_{des}\right)\pm \sqrt{{\left(k_{tr}+k_{ads}+k_{des}\right)}^{2}-4k_{tr}k_{des}}\right]/2$ are the eigenvalues of the linear equation system used to derive Equation (2). 

The molecular free energy Δ*G* can be computed using Equation (3):

(3) $$\Delta G=k_{B}Tln\frac{k_{ads}}{k_{des}}$$

where $k_{B}$ is the Boltzmann constant and *T* is the absolute temperature. 

The rate constants *k_tr_*, *k_ads_*, *k_des_*, as well as the free energy Δ*G* and the *R*^2^ parameter were obtained by fitting the experimental data. All experimental data show very good agreement with the fitted three-parameters model, as *R*^2^ values above 0.994 were obtained for all samples, except Resv@MCM-41 with a R^2^ value of 0.986.

### 3.3. Cytotoxicity, Apoptosis and Cell Cycle Analysis

Resv@MCM-48C, Resv@SBA-15-SH, Resv@SBA-15-Ph, Resv@SBA-15-NCO and Resv@MCF were selected for cell culture studies due to their favorable sustained release profiles. A549 non-small cell lung carcinoma, MDA-MB-231 triple negative breast cancer and human skin fibroblast (HSF) cell lines were used as an in-vitro model to evaluate the cytotoxicity of the newly developed mesoporous silica-based materials. To determine the half-maximal inhibitory concentration of the test agents (IC_50_), five different sample concentrations were applied to the cells and the SRB assay method was implemented. Cell viabilities were determined after 72 h of treatment and IC_50_ values were computed for resveratrol, carriers, and resveratrol-loaded carriers (Table 3).

**Figure 6:**
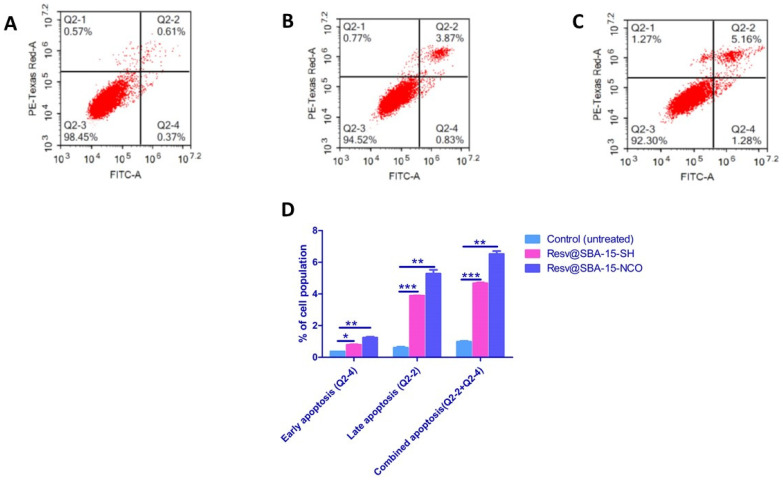
Apoptosis in A549 cells after 48 h exposure to Resv@SBA-15-SH and Resv@SBA-15-NCO samples. Cytograms showing annexin-V/Propidium Iodide-stained untreated A549 cells as negative control (**A**), cells treated with Resv@SBA-15-SH (**B**), cells treated with Resv@SBA-15-NCO (**C**) and representation of the apoptosis analysis results (**D**). Quadrant charts display Q2-1 (necrotic cells, AV-/PI+), Q2-2 (late apoptotic cells, AV+/PI+), Q2-3 (normal cells, AV-/PI-), Q2-4 (early apoptotic cells, AV+/PI-). Data represented is the average of triplicate experimental trials. The *p* values are denoted as: * ≤0.05, ** ≤0.005 and *** ≤0.0005.

Apoptosis assays were performed on the samples, which have demonstrated the highest cytotoxic effects on A549 cells (Resv@SBA-15-SH and Resv@SBA-15-NCO). The cells were treated for 48 h at their estimated IC_50_ value concentrations. Non-treated cells were considered the negative control (Table 4, Figure 6). 

**Figure 7:**
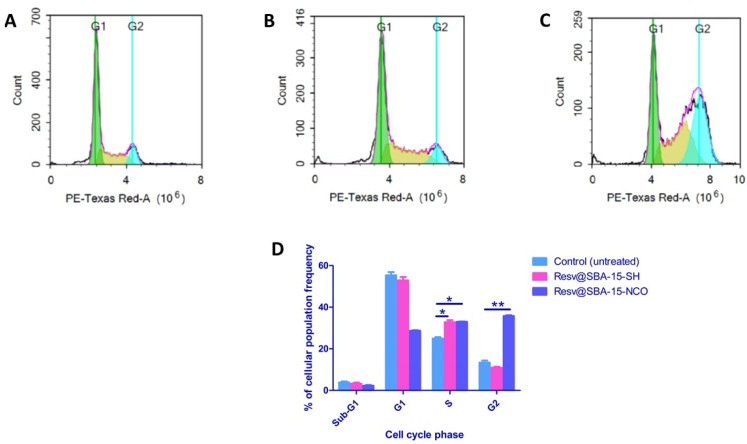
Cytogram representing cell cycle distribution of A549 cells after 48 h exposure of non-treated A549 cells (control) (**A**), A549 cells treated with Resv@SBA-15-SH (**B**), A549 cells treated with Resv@SBA-15-NCO S (**C**) and percentage of A549 cell population in various cell cycle phases (**D**); *p* values are denoted as: *  ≤0.05 and **  ≤0.005.

DNA flow cytometry was employed to determine the cell cycle kinetics of A549 lung cancer cell line pre-incubated for 48 h with Resv@SBA-15-SH and Resv@SBA-15-NCO at their IC_50_ concentrations of 26.1 μg/mL and 36.5 μg/mL, respectively (Table 5). The exact phase of the cell cycle in which the investigated samples interfere was determined by flow cytometry (Figure 7).

## 4. Discussion

### 4.1. Physico-Chemical Characterization

All FTIR spectra indicate the successful formation of the mesoporous silica carriers (Figure 1). The presence of the desired functional groups was also evidenced through FT-IR spectroscopy (Figure 1A). The FTIR analysis of the resveratrol–loaded carriers consist of the superposition of the silica and resveratrol spectra (Figure 1B), thus indicating the presence of the biological active compound. The main resveratrol vibrations in the 500–1500 cm^−1^ range, corresponding to C-H, C-C, C = C and C-O vibrations are present in all spectra of the resveratrol-loaded carriers. 

All functionalized carriers show similar organic groups content except SBA-15-NH_2_. The higher aminopropyl moieties content signifies that some polycondensed amine groups are still present despite the acid washing treatment [33]. All resveratrol-loaded materials have 15.5–20 wt.% resveratrol apart from the Resv@MCM-41 and Resv@SBA-Ph samples. This variation is likely explained by the difference in carrier hydrophobicity. The low pore size of MCM-41 and hydrophobic nature of the SBA-15-Ph carrier surface probably impede resveratrol adsorption, yielding a lower resveratrol content.

Nitrogen adsorption–desorption isotherms show that the adsorbed N_2_ volume is reduced for the resveratrol-loaded samples in comparison with that of carriers in all cases (Figure 1C, Figure S1, Supplementary Information). The specific surface area and total pore volume are also significantly decreased after loading the biological active compound, demonstrating its presence inside the mesopores (Table 1). The introduction of different organic groups through functionalization of SBA-15 decreases the total pore volume and specific surface area. The pore size distribution of the functionalized carriers shows a secondary peak at lower pore diameters, indicating the successful grafting of the organic groups onto the inner mesopore surface (Figure S2, Supplementary Information). The pore diameter of SBA-COOH is higher than that of SBA-15 while the pore size distribution is less monodisperse, which might be explained by a partial degradation of the silica framework during the strong acidic medium in which the hydrolysis reaction of SBA-15-CN took place [40]. The SBA-15-NH_2_ has lower pore diameter than SBA-15, probably due to the higher amount of functional groups in comparison with the other functionalized carriers (Table 1). 

The presence of well-defined small-angle XRD peaks for the functionalized SBA-15 samples indicates that the ordered mesopore structure was preserved after the chemical grafting of the organic groups (Figure S3, Supplementary Information). The lower intensity and broadness of the wide-angle resveratrol peaks indicates that it is adsorbed as a mixture of crystalline and amorphous phases inside the carrier mesopores. The crystallite size values (Table 2) are smaller than the carriers pore size (Table 1), which suggests that a small part of the bioactive molecule is able to crystallize into the pores of the carrier during the polyphenol loading, being nanoconfined into the mesoporore channels of the silica carrier.

The DSC data shows that most resveratrol-loaded samples have significantly reduced melting points with respect to the bulk polyphenol (Table 2). Only the Resv@SBA-15 sample has an endothermic event around the melting point of the bulk drug (265 °C) indicating the presence of both nanoconfined and interparticle crystalline phases (Figure 3). All other samples thus only contain crystalline resveratrol phase inside the silica mesopores. The Resv@MCM-41 melting point is lower than for SBA-15-type samples as expected from equation 1. Similarly, Resv@MCF has the highest melting point, as expected from the larger pore diameter of the silica carrier (Table 2). The inverse relationship between resveratrol melting point decrease, Δ*T*, and the pore diameter can be noticed for all samples, strongly suggesting that the biological active molecules are present only inside the mesopores of the carriers (Figure S4, Supplementary Information). Thus, the DSC analyses show the presence of only nanoconfined crystalline resveratrol for most samples [41]. The total pore volume decrease after loading is higher than expected purely from the weight fraction of the biological active molecule, indicating that the nanoconfined molecules are present inside the carrier mesopores. The heat of fusion values for the resveratrol–loaded pristine silica samples are lower than expected based on the weight fraction of the biological active molecules. Hence, a fraction of the adsorbed molecules must exist in an amorphous state. The percent of crystalline resveratrol varies between 2.2 and 30 wt.% depending on the sample, with the highest percent of crystallinity obtained for the Resv@MCF material possessing the largest pore diameter and pore volume (Table 1). The fraction of crystalline drug is directly correlated with the silica matrix average pore size (*p* = 0.044) and total pore volume (*p* = 0.016) (Supplementary Information, Table S1). The crystalline fraction is also inversely correlated with the specific surface area of the initial matrices (*p* = 0.034, Table S1). Thus, the amount of crystalline resveratrol is mainly explained by steric factors, with higher matrix pore volume and diameter resulting in higher crystalline fraction and with a higher surface area yielding more drug molecules adsorbed as an amorphous surface layer, resulting in a lower crystalline phase fraction. Our results are in accordance with the literature data showing that the resveratrol encapsulation into mesopores of pristine MSN has only led to the amorphization of a part of polyphenol molecules [42].

### 4.2. In Vitro Release Profiles and Kinetics Model

The model used to fit the experimental release data is a theoretical one, in contrast with most other empirical or semi-empirical models. The three-parameter model was shown to be applicable for the release of small organic molecules from mesoporous silica [43,44]. The main advantage of the three-parameter model comes from the fact that the kinetic parameters can be used to explain the release process. The diffusion process is significantly faster than the adsorption or desorption processes; as such, the release rate during the initial burst stage is proportional to the *k_tr_* constant. The burst stage finishes after most of the initially desorbed molecules are transported into the release medium. Afterwards, desorption becomes the rate limiting process and the sustained release rate is proportional to *k_des_*. Another consequence of the much higher diffusion is that the amount of biological active compound release during the burst stage is proportional to the free energy, *ΔG*, which shows the initial ratio of adsorbed to desorbed molecules (Figure S6, Supplementary Information). 

**Figure 8:**
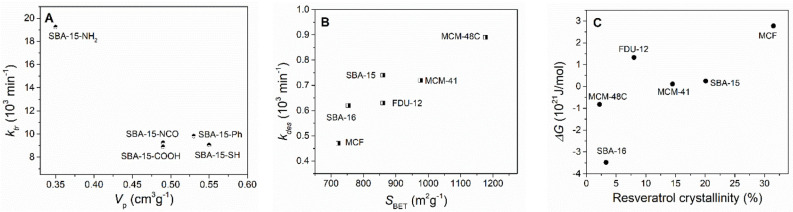
Correlation between: *k_tr_* and total pore volume for the resveratrol–loaded materials containing functionalized carriers (**A**); *k_des_* and specific surface area for the samples containing pristine silica carriers (**B**) and ΔG and the resveratrol crystallinity computed from DSC analyses for materials containing pristine silica carriers (**C**).

The diffusion and transport rate constant, *k_tr_*, is proportional to the release rate during the burst stage, under the assumption that *k_tr_* is significantly higher than the rates of adsorption and desorption. The fitted data show that indeed *k_tr_* is generally an order of magnitude higher than *k_ads_* and *k_des_ (*Table 6). All resveratrol–loaded carriers exhibit higher burst release rates than pure resveratrol dissolution rate. The highest diffusion rate is obtained for Resv@SBA-15-NH_2_, which might be explained by the lower pore volume after loading, resulting in a shorter diffusion length into the external release medium. This is supported by the inversely proportional correlation between the diffusion and transport rate constant and the remaining pore volume of the resveratrol-loaded materials containing pristine or functionalized SBA-15 carriers (Figure 8A, *p* = 0.009). The highest *k_tr_* value was obtained for Resv@SBA-16 in the case of the pristine carriers, although a correlation between pore volume and diffusion rate is not apparent. The highest diffusion and transport rate might be related to the cubic mesopore arrangement of the unfunctionalized SBA-16, which also results short diffusion lengths for the resveratrol molecules.

The desorption rate constant, *k_des_*, is proportional to the release rate during the sustained release regime. Desorption becomes the rate limiting process after most of the initially desorbed molecules have been transported into the release medium. Interestingly, the desorption rate constant is proportional to the specific surface area of the carriers in the case of the pristine silica samples (Figure 8B, *p* = 0.012). The desorption rate is also inversely correlated to the average pore diameter of both the pristine carriers and the drug-loaded materials (*p* = 0.042 and 0.045, respectively). The highest desorption rate can be noticed for the Resv@SBA-15-COOH sample, followed by Resc@SBA-15-NCO. The *k_des_* increase for these functional groups might be explained as repulsive or diminished attractive supermolecular interactions between the functional organic groups and resveratrol molecules in comparison with pristine silica. 

A higher amount of resveratrol dissolved in PBS pH 6.8 after 24 h was observed in the case of MCM-48C silica (16.21 ± 0.05 µg/mL), which had amorphous polyphenol into the mesopores, than for pure resveratrol (14.43 ± 0.00 µg/mL) or other pristine silica matrices with larger pores (SBA-16, SBA-15, FDU-12 or MCF) or mesopores forming long channels (MCM-41). The functionalization of SBA-15 silica surface with hydrophilic groups (isocyanate, carboxyl or aminopropyl) favored a higher cumulative drug release in PBS pH 6.8 after 24 h and thus, a higher amount of resveratrol dissolved after 24 h (in the range of 14.78–16.73 µg/mL) than in the case of pure resveratrol or when resveratrol was encapsulated in silica functionalized with more hydrophobic groups such as phenyl or mercaptopropyl (Table 6). 

The Δ*G* free energy parameter combines the effects of the adsorption and desorption rate constants. This parameter denotes the fraction of initially desorbed polyphenol molecules, and it is proportional to the resveratrol fraction release during the burst stage with respect to total biologically active compound amount. The Δ*G* parameter thus indicates the position of the inflection point between the burst and sustained release stages on the *y*-axis. Smaller (and negative) Δ*G* values indicate less resveratrol released during the burst stage. The free energy parameter increases with the increase of the crystalline resveratrol content in the case of the pristine silica carriers (Figure 8C, *p* = 0.084). This correlation can be explained by the fact that a higher Δ*G* parameter indicates that more drug molecules are desorbed from the silica pore surface at the start of the experiment. Since the adsorbed molecules are likely in an amorphous phase, a higher Δ*G* value will result in a lower amorphous phase fraction, which can be correlated with a higher crystalline phase content. An amorphous drug phase usually has higher solubility and dissolution rate than its crystalline counterpart, as the supermolecular interactions in the amorphous phase are weaker. However, in the case of drug-loaded mesoporous silica, the amorphous drug phase also makes supermolecular interactions with the matrix pore surface, which might reduce the apparent solubility of this amorphous phase. In the case of the functionalized carriers, the free energy parameter is strongly influenced by the nature of the functional group grafted on carrier surface, the lowest Δ*G* value being obtained for Resv@SBA-15-SH sample. 

### 4.3. Cytotoxicity, Apoptosis and Cell Cycle Analysis

Resveratrol-loaded mesoporous silica materials were highly effective as cytotoxic agents on A549 non-small cell lung carcinoma and MDA-MB-231 breast cancer cells, with the IC_50_ values ranging from 17.18–49.5 µg/mL. Pure resveratrol had an IC_50_ value of 6.7 and 6.34 µg/mL against A549 and MDA-MB-231, respectively (Table 2). The IC_50_ of the unloaded carriers was higher than 500 µg/mL which points to their safety, as they did not decrease the cell viability except when loaded with the desired drug. The Resv@SBA-15-SH and Resv@SBA-15-NCO samples possessed the most potent anticancer activity against A549 cells as denoted by the lowest IC_50_ values, of 26.06 and 36.5 µg/mL, respectively. Marinheiro et al. reported that A375 melanoma cells are more sensitive to either resveratrol-loaded MSN formulation or free resveratrol than MNT-1 cells. Hence, they obtained IC_50_ values of 21.1 μg/mL and 29.5 μg/mL for A375 cell line and MNT-1 cells after 48 h and 72 h of exposure, respectively [42]. Recently, Lin et al. found that resveratrol inhibited proliferation, migration, and invasion while promoting apoptosis in human gastric cancer HGC-27 and AGS cells, and alleviated tumor size in vivo, greater effects being observed for resveratrol-loaded MSN than in the case of free polyphenol [45]. An improved antiproliferative potential of resveratrol when is loaded on phosphonate modified MSN on prostate cancer PC3 cells with an IC_50_ of 7.15 µM as compared to 14.86 µM of free resveratrol was reported [28]. 

Concerning the breast cancer cell line MDA-MB-231, a different cytotoxic effect from the pattern observed with A549 cells was observed. For the MDA-MB-231 breast cancer cells, the two lowest IC_50_ values were 17.18 ± 0.96 µg/mL and 19.30 ± 1.08 µg/mL, which were the result of exposure to Resv@SBA-15-Ph and Resv@SBA-15-NCO, respectively. Our results are corroborated by recent publications that report the agonistic effect of resveratrol against the same cell line used in the present study, when added simultaneously with the chemotherapeutic cisplatin [46]. The possible use of the developed loaded carriers against lung adenocarcinoma, and the aggressive triple negative breast adenocarcinoma cell lines, each with different cytotoxic effects to each type of cells, is worthy of mention. 

Our data analysis showed that the late apoptotic sub-population (AV+/PI+) was 3.86% in cells treated with Resv@SBA-15-SH which was 6-fold more than that of the untreated cells (*p* = 0.0003) (Table 4, Figure 5). A higher effect was observed in the cells treated with Resv@SBA-15-NCO, where the late apoptotic sub-population was found to be 5.283% which is about 9-fold more that of the untreated cells (*p* = 0.003) (Table 4, Figure 5). The apoptosis assay results pointed towards a possible cellular mechanism of action for the two examined resveratrol-loaded samples. Resv@SBA-15-SH and Resv@SBA-15-NCO caused a higher fraction of A549 lung cancer cells to enter late apoptosis. 

Cell treatment with Resv@SBA-15-SH and Resv@SBA-15-NCO elicited an increase in the percent cells sub-population undergoing early apoptosis (AV+/PI−) by two (*p* = 0.0069) and three (*p* = 0.0041) folds, respectively (Table 4, Figure 5). Moreover, analysis of the combined apoptotic phases altogether after exposure to both formulations showed that Resv@SBA-15-SH and Resv@SBA-15-NCO were able to increase the average percentage of cells undergoing apoptosis by five (*p* < 0.0001) and seven (*p* = 0.0012) folds, respectively (Table 3, Figure 5). The selected resveratrol-loaded samples thus demonstrated significant increase in the induction of apoptosis as compared to the untreated lung cancer cells.

Cell cycle analysis results showed an observable S-phase sequestration with about 32% increase in the frequency of S-phase population (*p* = 0.008) upon exposure to Resv@SBA-15-SH (*p* = 0.008) and Resv@SBA-15-NCO (*p* = 0.0084) at their respective estimated IC_50_ values (Table 4, Figure 6). Resv@SBA-15-NCO caused about 3-fold increase in cell retention the G2-phase (*p* = 0.0023) (Table 4, Figure 6). Both samples have strong ability to interfere with the cells in their ploidy stage (4n), i.e., to interfere with the DNA synthesis and chromosomal replication. The results are in strong alignment with Zheng et al. work who reported that administration of high dose of resveratrol could deplete the dNTPs and thereby interfere with DNA synthesis in the S phase, while administration of low dose resveratrol resulted sequestration of cells at the S phase and slowed down cells’ progression to the following phase of the cell cycle [47]. The Resv@SBA-15-NCO material has shown an additional effect of trapping cells at the G2-phase, which in turn stops their progression to the mitotic phase (M-phase) and hinders the process of cell division. The results are presented in Table 5 and Figure 6. Accordingly, some nano formulations not only excelled at improving resveratrol’s action on drug delivery properties, but also demonstrated potential therapeutic effects on the cellular level.

## 5. Conclusions

Resveratrol release profiles showed enhanced dissolution rates when it was encapsulated in mesoporous silica-type carriers. The burst release rate was higher for all samples containing mesoporous silica. Most materials apart from the Res@SBA-16 and Resv@SBA-15-SH exhibit higher cumulative release than pure resveratrol. The highest cumulative release after 24 h is obtained for the SBA-15-COOH carrier. 

The interplay between the mesoporous silica carrier textural properties, amount of resveratrol-loaded and physical state and selection of functional groups can therefore be used to tailor the release profiles and thus its dissolution rate.

Biological evaluation of proposed mesoporous silica carriers demonstrated a high safety profile on tested cell lines. The resveratrol-loaded mesoporous silica-type carriers showed remarkable anticancer effects on A549 non-small cell lung cancer and MDA-MB-231 breast cancer cells. These findings provide a further clue for proposed formulations suitability as anticancer agents with better pharmacokinetic profile and potent biological activity.

## Figures and Tables

**Table 1:** Molar ratio of silica to organic groups (SiO_2_/OG), resveratrol content (Resv), specific surface area (S_BET_), total mesopore volume (V_p_) and BJH pore diameters computed from the adsorption (d_ads_) and desorption (d_des_) branches of the isotherm.

Sample	Carriers					Resveratrol-Loaded Samples				
	SiO_2_/OG * (mol)	S_BET_ (m^2^g^−1^)	V_p_ (cm^3^g^−1^)	d_des_ (nm)	d_ads_ (nm)	Resv (wt.%)	S_BET_ (m^2^g^−1^)	V_p_ (cm^3^g^−1^)	d_des_ (nm)	d_ads_ (nm)
MCM-41	-	976	0.77	2.7	2.7	14.8	814	0.62	2.5	2.5
MCM-48C	-	1177	0.91	2.7	2.7	20.1	492	0.42	2.7	2.7
SBA-16	-	753	0.71	3.5	5.1	15.6	287	0.27	3.5	5.1
FDU-12	-	860	0.88	3.7	9.0	18.0	345	0.45	3.7	9.0
MCF	-	722	2.38	11.4	33.5	18.0	487	2.06	11.4	33.5
SBA-15	-	860	1.07	6.3	8.1	18.8	391	0.56	5.8	7.4
SBA-15-COOH	13.5	386	0.77	7.4	10.0	16.4	234	0.49	6.8	9.0
SBA-15-NCO	14.3	541	0.76	6.3	8.1	19.7	318	0.49	5.8	7.4
SBA-15-NH_2_	6.0	451	0.59	5.4	6.8	20.2	208	0.35	5.1	6.8
SBA-15-Ph	25.0	732	0.95	6.3	7.4	13.8	347	0.53	6.3	8.1
SBA-15-SH	17.5	808	0.95	6.3	8.2	20.9	371	0.55	6.3	8.1

* OG—organic groups.

**Table 2:** Melting points (m.p.) and heat of fusion (Δ*H*) for the resveratrol-loaded carriers, computed crystalline and amorphous phase contents, as well as crystallite size determined from XRD.

Sample	Resv. (wt.%)	m.p. (°C)	Δ*H* (J g^−1^)	Content of Crystalline Phase * (wt.%)	Content of Amorphous Phase ** (wt.%)	Crystallite Size (nm)
Resveratrol	100	266	−250	100	0	30.59
Resv@MCM-41	14.8	178	−5.38	14.5	85.5	-
Resv@MCM-48C	20.1	209	−1.11	2.2	97.8	-
Resv@SBA-16	15.6	183	−1.27	3.3	96.7	-
Resv@FDU-12	18.0	195	−3.59	8.0	92.0	-
Resv@MCF	18.0	230	−14.17	31.5	68.5	22.50
Resv@SBA-15	18.8	181	−9.45	20.1	79.9	2.71
Resv@SBA-15-COOH	16.4	190	−10.16	24.8	75.2	2.04
Resv@SBA-15-NCO	19.7	199	−22.91	46.5	53.5	3.42
Resv@SBA-15-NH_2_	20.2	198	−1.44	2.9	97.1	-
Resv@SBA-15-SH	20.9	204	−4.99	9.6	90.4	-

* $Content\;of\;crystalline\;phase=100\frac{\Delta H\mathrm{sample}}{w_{Resv}\Delta H\mathrm{resv}}$, where $w_{Resv}$ represents the resveratrol weigh fraction. ** Content of amorphous phase = 100 – Content of crystalline phase.

**Table 3:** Cytotoxicity of the resveratrol-loaded samples against non-small cell lung carcinoma (A549), triple negative breast cancer (MDA-MB231) and human skin fibroblast (HSF) cell lines after 72 h treatment.

Sample	IC_50_ on A-549 Cell Line * (µg/mL)	IC_50_ on MDA-MB231 Cell Line * (µg/mL)	IC_50_ on HSF Cell Line * (µg/mL)
Resveratrol	6.7 ± 0.38	6.34 ± 0.37	7.0 ± 0.35
SBA-15-SH	>500	>500	>500
Resv@SBA-15-SH **	26.1 ± 1.11	35.56 ± 1.29	26.1± 1.09
SBA-15-Ph	>500	>500	>500
Resv@SBA-15-Ph	37.6 ± 1.29	17.18 ± 0.96	31.4 ± 1.85
MCM-48C	>500	>500	>500
Resv@MCM-48C	45.3 ± 1.42	30.80 ± 1.93	37.2 ± 1.32
MCF	>500	>500	>500
Resv@MCF	49.5 ± 2.16	23.12 ± 1.02	33.5 ± 1.49
SBA-15-NCO	>500	>500	>500
Resv@SBA-15-NCO **	36.5 ± 1.14	19.30 ± 1.08	32.4 ± 1.28

* IC_50_ values are the mean of triplicate experimental runs ± standard deviation. ** The two nano-carrier formulations with the lowest IC_50_ values that are the most potent on A549.

**Table 4:** Apoptosis assay of A549 cells after treatment with the two most potent resveratrol-loaded materials for 48 h.

Apoptotic Stage	Percent Cell Population *		
	Negative Control	Resv@SBA-15-SH	Resv@SBA-15-NCO
Late apoptosis (Q2-2)	0.613 ± 0.095	3.89 ± 0.026	5.283 ± 0.419
Early apoptosis (Q2-4)	0.367 ± 0.006	0.790 ± 0.061	1.240 ± 0.096
Combined apoptosis (Q2-2 + Q2-4)	0.980 ± 0.1	4.680 ± 0.072	6.523 ± 0.323

* Data presented herein as the mean of triplicate independent runs ± standard deviation.

**Table 5:** Cell cycle analysis of A549 cells treated with Resv@SBA-15-SH and Resv@SBA-15-NCO for 48 h.

Cell Cycle Phases	Control	Resv@SBA-15-SH (26.1 μg/mL)	Resv@SBA-15-NCO (36.5 μg/mL)
Freq Sub-G1	3.823 ± 0.891	3.2 ± 0.97	2.297 ± 0.47
Freq G1	55.31 ± 2.708	52.88 ± 2.871	28.66 ± 0.427
Freq S	24.91 ± 1.267	32.75 ± 1.737	32.97 ± 0.227
Freq G2	13.35 ± 1.75	10.94 ± 0.75	35.77 ± 0.59

Data are presented as the mean of triplicate experimental runs ± standard deviation. Freq is denoted as frequency or the number of cells at different phases of the cell cycle (Sub-G1, G1, S and G2 phases).

**Table 6:** Resveratrol amount dissolved after 24 h in PBS pH 6.8 at 37 °C, free energy, rate constants, and R^2^ values of three-parameter kinetic model.

Sample	Resv Dissolved after 24 h (µg/mL)	Δ*G* (10^21^ J/mol)	*k_tr_* (10^3^ min^−1^)	*k_des_* (10^3^ min^−1^)	*k_ads_* (10^3^ min^−1^)	*R* ^2^
Resv	14.43 ± 0.00	−1.36 *	7.16 *	0.72 *	0.99 *	1.000 *
Resv@MCM-41	11.53 ± 0.06	0.11	8.66	0.72	0.70	0.986
Resv@MCM-48C	16.21 ± 0.05	−0.82	12.37	0.89	1.08	0.998
Resv@SBA-16	10.52 ± 0.03	−3.48	16.62	0.62	1.40	0.996
Resv@FDU-12	14.63 ± 0.05	1.33	8.11	0.74	0.54	0.997
Resv@MCF	14.42 ± 0.10	2.77	8.63	0.47	0.24	0.994
Resv@SBA-15	14.34 ± 0.09	0.25	9.03	0.63	0.59	0.995
Resv@SBA-15-COOH	15.87 ± 0.08	1.79	8.90	2.59	1.70	0.999
Resv@SBA-15-NCO	16.73 ± 0.05	−1.17	9.26	1.3	1.71	0.999
Resv@SBA-15-NH_2_	14.78 ± 0.08	−0.69	19.24	0.54	0.64	0.998
Resv@SBA-15-Ph	11.63 ± 0.05	1.27	9.83	1.13	0.84	0.999
Resv@SBA-15-SH	13.31 ± 0.06	−2.01	9.06	0.47	0.75	0.998

* The three-parameters model was also applied for resveratrol dissolution, although it has no physical signification.

## Data Availability

Not applicable.

## Supplementary Materials

The following are available online at https://www.mdpi.com/article/10.3390/pharmaceutics14010203/s1. Figure S1. Nitrogen adsorption-desorption isotherms of mesoporous carriers and resveratrol-loaded carriers: MCF and Resv@MCF (**A**); FDU-12, SBA-16 and Resv@FDU-12, Resv@SBA-16 (**B**); MCM-48 and Resv@MCM-48 (**C**); MCM-41 and Resv@MCM-41 (**D**); SBA-15-COOH and Resv@SBA-15-COOH; Figure S2. Pore size distribution curves determined from nitrogen adsorption-desorption isotherms for SBA-15-type carriers (**A**) and resveratrol-loaded SBA-15-type carriers (**B**); Figure S3. Small–angle XRD patterns of pristine and functionalized SBA-15 carriers; Figure S4. DSC traces of resveratrol-loaded samples containing functionalized carriers; Figure S5. Representation of the three-parameter model; Table S1. Correlation coefficients and *p*-values for the drug-loaded samples obtained using pristine silica carriers; Table S2. Correlation coefficients and *p*-values for the drug-loaded samples obtained using functionalized silica carriers.
