# Supplementary material for: Resveratrol Encapsulation and Release from Pristine and Functionalized Mesoporous Silica Carriers
Source: Pharmaceutics. 2022 Jan 16;14(1):203. doi: 10.3390/pharmaceutics14010203 (PMC8780957; doi:10.3390/pharmaceutics14010203)
Supplement: Supplementary file 1 [file pharmaceutics-14-00203-s001.zip › pharmaceutics-1516858-supplementary.pdf]

pharmaceutics

MDPI

Supplementary Materials: Resveratrol Encapsulation and Release from Pristine and Functionalized Mesoporous Silica Carriers

Simona Ioniţă, Daniel Lincu, Raul-Augustin Mitran, Laila Ziko, Nada K. Sedky, Mihaela Deaconu, Ana-Maria Brezoiu, Cristian Matei [1] and Daniela Berger

Characterization of materials

Nitrogen adsorption - desorption isotherms

Nitrogen adsorption – desorption isotherms were recorded for all samples and the corresponding pore size distribution curves were determined from corresponding isotherm (Figure S1).

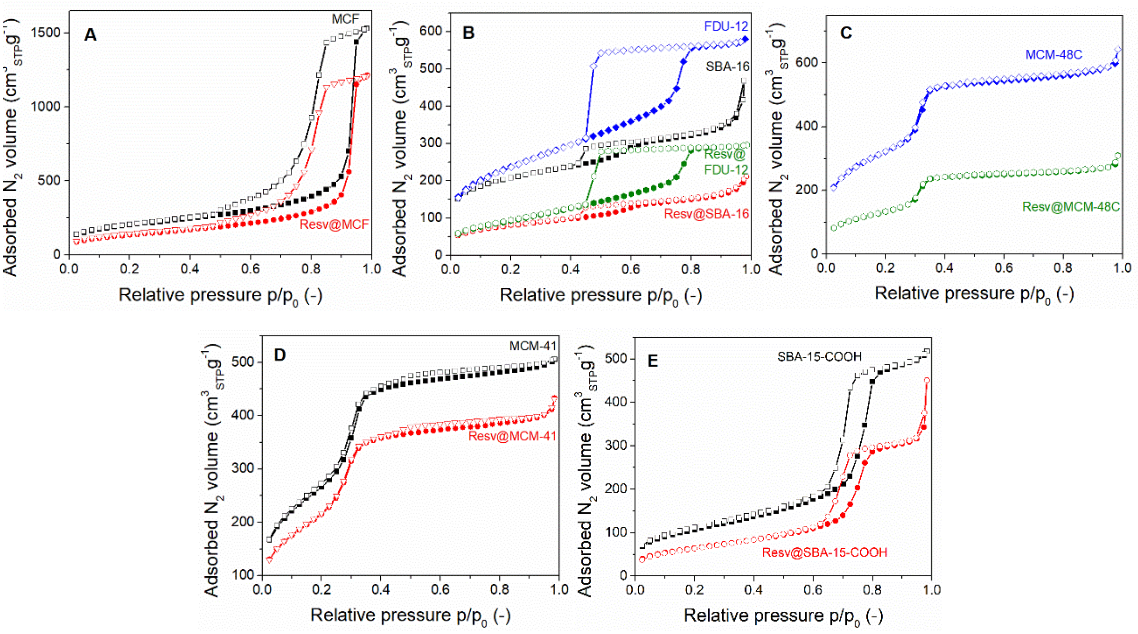

Figure S1. Nitrogen adsorption-desorption isotherms of mesoporous carriers and resveratrol-loaded carriers: MCF and Resv@MCF (A); FDU-12, SBA-16 and Resv@FDU-12, Resv@SBA-16 (B); MCM-48 and Resv@MCM-48 (C); MCM-41 and Resv@MCM-41 (D); SBA-15-COOH and Resv@SBA-15-COOH (E).

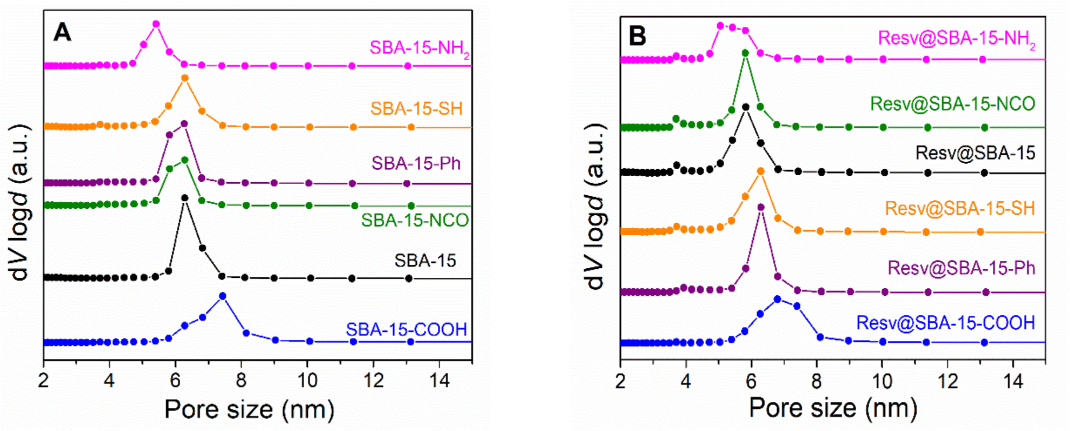

Figure S2. Pore size distribution curves determined from nitrogen adsorption-desorption isotherms for SBA-15-type carriers (**A**) and resveratrol-loaded SBA-15-type carriers (**B**).

X-ray diffraction

The small-angle XRD patterns of pristine and functionalized SBA-15 carriers exhibit the characteristic (110) and (200) Bragg reflections of the *P6mm* hexagonal space group (Figure S3).

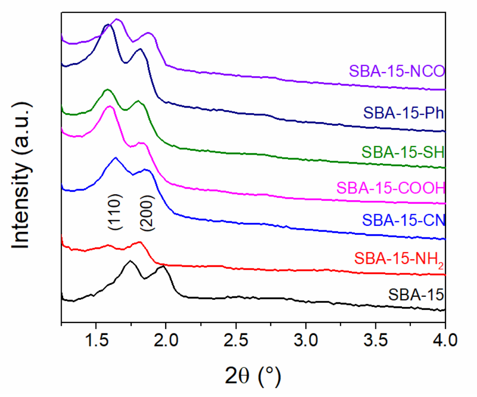

Figure S3. Small–angle XRD patterns of pristine and functionalized SBA-15 carriers.

DSC analysis

The DSC traces of resveratrol–loaded functionalized SBA-15 carriers are presented in Figure S4.

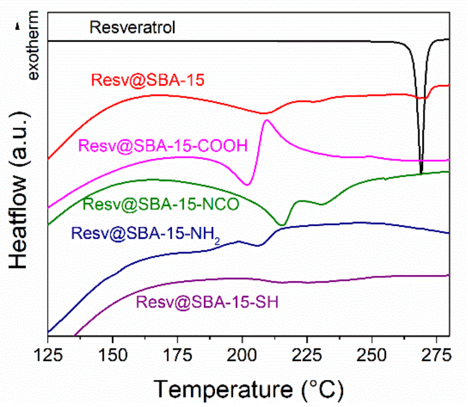

Figure S4. DSC traces of resveratrol – loaded samples containing functionalized carriers.

Release profiles

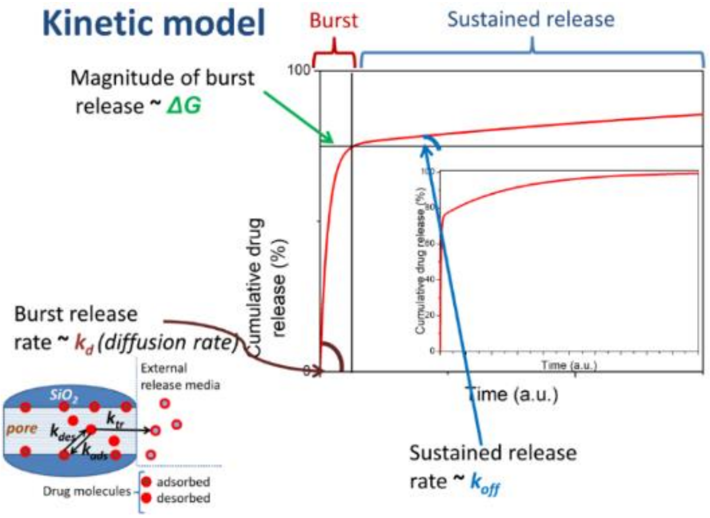

Figure S5. Representation of the three-parameter model.

Correlation of model parameters to textural properties

Two correlation studies between the fitted parameters of the theoretical kinetics model and the physico-chemical parameters of the carriers and resveratrol-loaded samples have been performed using Microsoft Excel. The two correlation studies were carried out on the materials containing pristine mesoporous silica (MCM-41, MCM-48C, SBA-16, FDU-12, MCF, SBA-15) or on the functionalized samples, including the parent SBA-15 (SBA-15, SBA-15-COOH, SBA-15-NCO, SBA-15-NH_2, SBA-15-Ph, SBA-15-SH). The correlation coefficients *r* take values between -1 and 1, with values close to 1 indicating direct correlation and values close to -1 indicating inverse correlation (Table S1, Table S2). The *p*-values were computed for each pair of model parameter – physico-chemical property.

Table S1. Correlation coefficients, *r* and *p*-values for the drug-loaded samples obtained using pristine silica carriers.

				Carriers				Resveratrol-loaded samples			
	ΔH (*J g-1*)	*Crystalline* (*% wt.*)	*Amorphous* (*% wt.*)	S_{BET} (m^2g^{-1})	V_p (cm^3g^{-1})	*d des* (*nm*)	*d ads* (*nm*)	S_{BET} (m^2g^{-1})	V_p (cm^3g^{-1})	*d des* (*nm*)	*d ads* (*nm*)
ΔG	-0.75 (p = 0.084)	0.75 (p = 0.085)	-0.75 (p = 0.085)	-0.15 (p = 0.778)	0.71 (p = 0.114)	0.65 (p = 0.163)	0.69 (p = 0.129)	0.26 (p = 0.615)	0.73 (p = 0.099)	0.65 (p = 0.166)	0.69 (p = 0.132)
k_{tr}	0.6 (p = 0.205)	-0.62 (p = 0.192)	0.62 (p = 0.192)	-0.01 (p = 0.983)	-0.38 (p = 0.461)	-0.36 (p = 0.489)	-0.35 (p = 0.493)	-0.43 (p = 0.399)	-0.43 (p = 0.394)	-0.34 (p = 0.516)	-0.34 (p = 0.504)
k_{des}	0.78 (p = 0.069)	-0.78 (p = 0.068)	0.78 (p = 0.068)	**0.91 (p = 0.012)**	-0.7 (p = 0.123)	**-0.83 (p = 0.042)**	-0.78 (p = 0.069)	0.17 (p = 0.749)	-0.7 (p = 0.118)	**-0.82 (p = 0.045)**	-0.77 (p = 0.073)
k_{ads}	**0.83 (p = 0.039)**	**-0.84 (p = 0.039)**	**0.84 (p = 0.039)**	0.28 (p = 0.586)	-0.69 (p = 0.129)	-0.69 (p = 0.132)	-0.69 (p = 0.132)	-0.25 (p = 0.627)	-0.72 (p = 0.108)	-0.67 (p = 0.143)	-0.68 (p = 0.139)

Crystalline (% wt.)	-	-	-	**-0.52 (p = 0.034)**	**0.85 (p = 0.016)**	**0.90 (p = 0.044)**	0.82 (p = 0.658)	**0.23 (p = 0.023)**	**0.87 (p = 0.022)**	**0.88 (p = 0.05)**	0.81 (p = 0.085)

Table S2. Correlation coefficients, *r* and *p*-values for the drug-loaded samples obtained using functionalized silica carriers.

			Carriers				Resveratrol-loaded samples			
	Resv.(% wt.)	SiO_2/OG (mol)	S_{BET} (m^2g^{-1})	V_p (cm^3g^{-1})	*d des* (*nm*)	*d ads* (*nm*)	S_{BET} (m^2g^{-1})	V_p (cm^3g^{-1})	*d des* (*nm*)	*d ads* (*nm*)
ΔG	**-0.87 (p = 0.025)**	0.27 (p = 0.6)	-0.26 (p = 0.614)	0.1 (p = 0.857)	0.56 (p = 0.246)	0.41 (p = 0.423)	-0.24 (p = 0.651)	0.08 (p = 0.875)	0.47 (p = 0.343)	0.53 (p = 0.278)
k_{tr}	0.30 (p = 0.568)	-0.7 (p = 0.119)	-0.43 (p = 0.399)	-0.72 (p = 0.107)	-0.74 (p = 0.089)	-0.64 (p = 0.174)	-0.66 (p = 0.152)	**-0.92 (p = 0.009)**	-0.77 (p = 0.071)	-0.65 (p = 0.164)
k_{des}	-0.52 (p = 0.289)	0.05 (p = 0.92)	-0.63 (p = 0.178)	-0.22 (p = 0.67)	**0.84 (p = 0.034)**	0.81 (p = 0.052)	-0.44 (p = 0.388)	0.03 (p = 0.958)	0.67 (p = 0.144)	0.74 (p = 0.091)
k_{ads}	-0.16 (p = 0.757)	-0.05 (p = 0.928)	-0.63 (p = 0.181)	-0.35 (p = 0.502)	0.65 (p = 0.159)	0.67 (p = 0.147)	-0.34 (p = 0.516)	0 (p = 0.994)	0.46 (p = 0.364)	0.48 (p = 0.339)
